# 5,6-Di­methyl­benzo[*d*][1,3]oxatellurole

**DOI:** 10.1107/S2414314623010763

**Published:** 2024-01-05

**Authors:** Samantha Ponzo, Alanna Turner, Frank R. Fronczek, Thomas Junk

**Affiliations:** a Department of Chemistry, Lafayette, LA 70403, USA; b Department of Chemistry, Louisiana State University, Baton Rouge, LA 70803, USA; University of Aberdeen, United Kingdom

**Keywords:** tellurium, heterocyclic, organotellurium, oxatellurole, crystal structure

## Abstract

The structure of the title compound, C_9_H_10_OTe, at 100 K has ortho­rhom­bic (*P*2_1_2_1_2) symmetry with two independent mol­ecules in the asymmetric unit (*Z*′ = 2). The mol­ecules are folded along their Te⋯O axes, with their Te–C–O planes angled at an average of 25.1° with respect to the remaining non-H atoms, which are almost coplanar (average deviation from planarity = 0.04 Å). A Hirshfeld plot shows weak inter­molecular inter­actions between the two Te atoms located in each asymmetric mol­ecule, with a Te⋯Te distance of 3.7191 (4) Å. The structure is strongly pseudosymmetric to the space group *Pccn* with *Z*′ = 1. The crystal chosen for data collection was found to be was an inversion twin.

## Abstract 2

The title compound represents the first reported example of a [1,3]oxatellurole, prepared in three steps from 3,4-di­methyl­phenol. Both independent mol­ecules are folded along their Te⋯O axes, with an average angle φ = 25.1° between the Te–C–O planes and the remaining non-hydrogen atoms. A Hirshfeld plot indicates a weak inter­molecular inter­action between the two tellurium atoms in the asymmetric unit.

## Graphical abstract

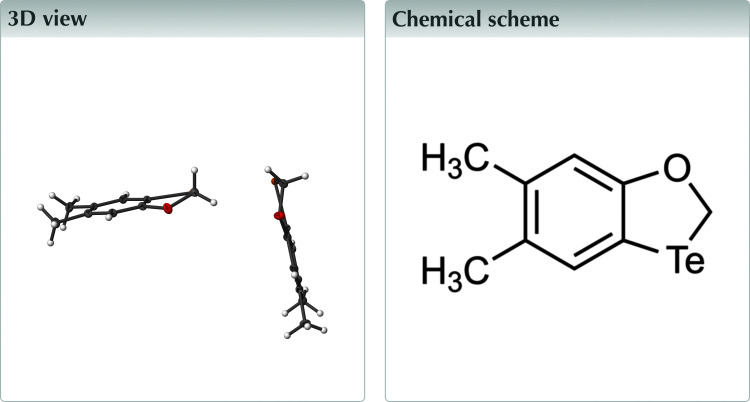

## Structure description

**Figure 1:**
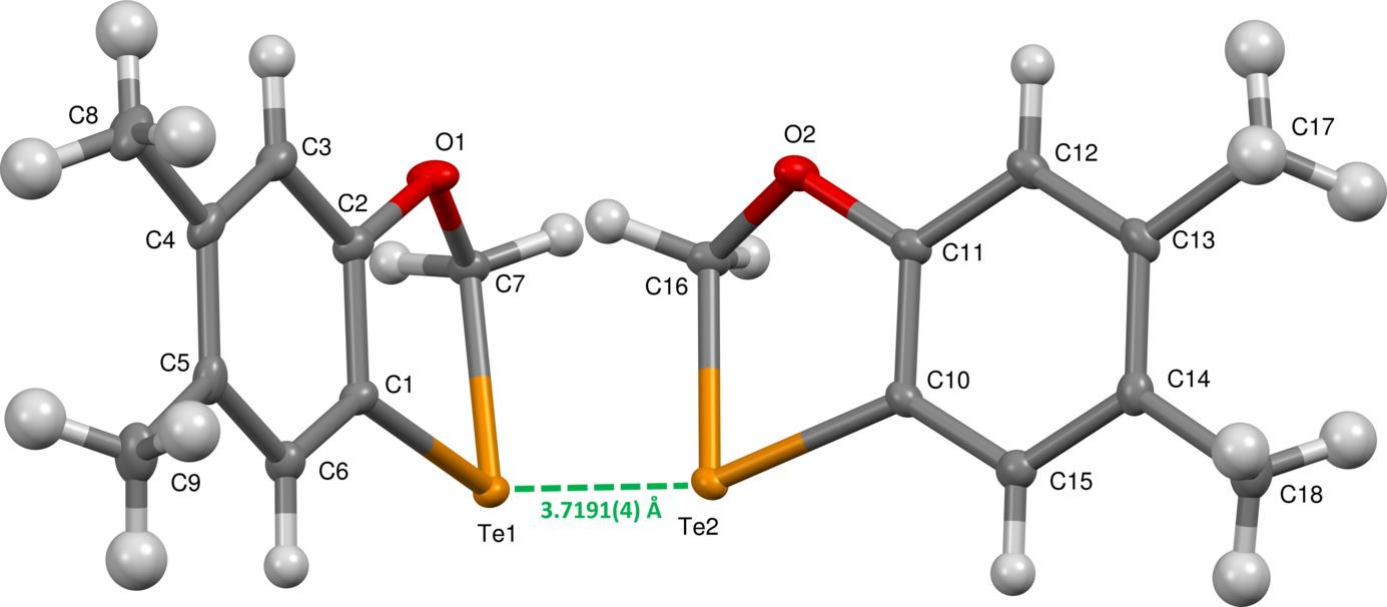
The asymmetric unit of the title compound with 50% displacement ellipsoids.

Tellurium/oxygen-containing heterocycles have received significant attention as potent enzyme inhibitors and anti­oxidants. Thus, organotelluroxetanes inhibit cysteine proteases (Persike *et al.*, 2008 ▸) while derivatives of 1,3,2-dioxatellurolane inhibit IL-1 β converting enzyme (Brodsky *et al.*, 2007 ▸; Ba *et al.*, 2010 ▸) and proteases (Albeck *et al.*, 1998 ▸). Deriv­atives of [1,2]oxatellurole act as gluta­thione peroxidase mimetics (Back *et al.*, 2005 ▸) while octa-*O*-bis-(*R*,*R*)-tartarate ditellurane (‘SAS’) provides pro-apoptotic signaling in drug-resistant multiple myeloma (Zigman-Hoffman *et al.*, 2021 ▸). [1,4]Oxatelluranes have been known for over seventy years (Farrar & Gulland, 1945 ▸). In contrast, [1,3]oxatelluroles have remained unknown, making the title compound, 5,6-di­methyl­benzo[*d*][1,3]oxatellurole, C_9_H_10_OTe, the first member of this class: the two mol­ecules in the asymmetric unit are shown in Fig. 1 ▸.

Furthermore, a search of the Cambridge Structural Database (May 2021 update; Groom *et al.*, 2016 ▸) for [1,3]oxa­seleno­les and [1,3]oxa­thio­les indicates a paucity of such structures as well. One [1,3]oxa­selenole (Laitalainen *et al.*, 1983 ▸) and thirteen sulfur congeners are known, such as the structurally similar 6,6-dimethyl-[1,3]dioxolo[4′,5′:4,5]benzo[1,2-*d*][1,3]oxa­thiole-8-carbaldehyde (Wessig *et al.*, 2021 ▸). Reported derivatives of selena­fulvalene and tetra­tellura­fulvalene (Kojima *et al.*, 2004 ▸; Carroll *et al.*, 1982 ▸) bear only limited structural resemblance to the title compound, since these mol­ecules are almost planar due to the absence of an *sp* ^3^-hybridized carbon atom in the heteroaromatic ring. The non-planarity in the title compound accommodates near-tetra­hedral angles at the bridging carbon atom [Te1—C7—O1 = 108.27 (19), Te2—C16—O2 = 108.16 (19)°] for the two independent mol­ecules; the C1—Te1—C7 and C10—Te2—C16 angles are 78.40 (11) and 78.24 (11)°, respectively.

**Figure 2:**
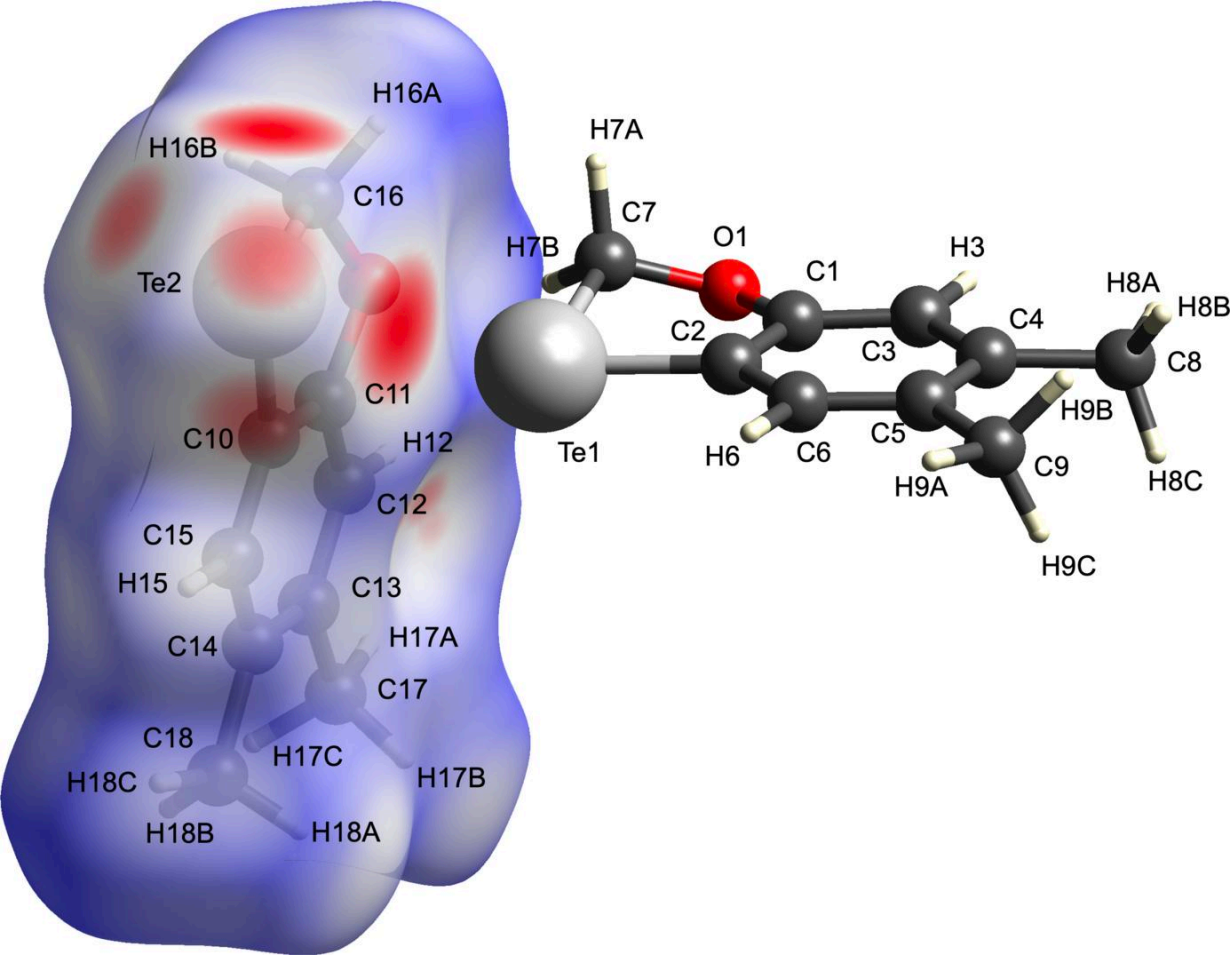
The Hirshfeld surface of the title compound mapped over *d*_norm_.

**Figure 3:**
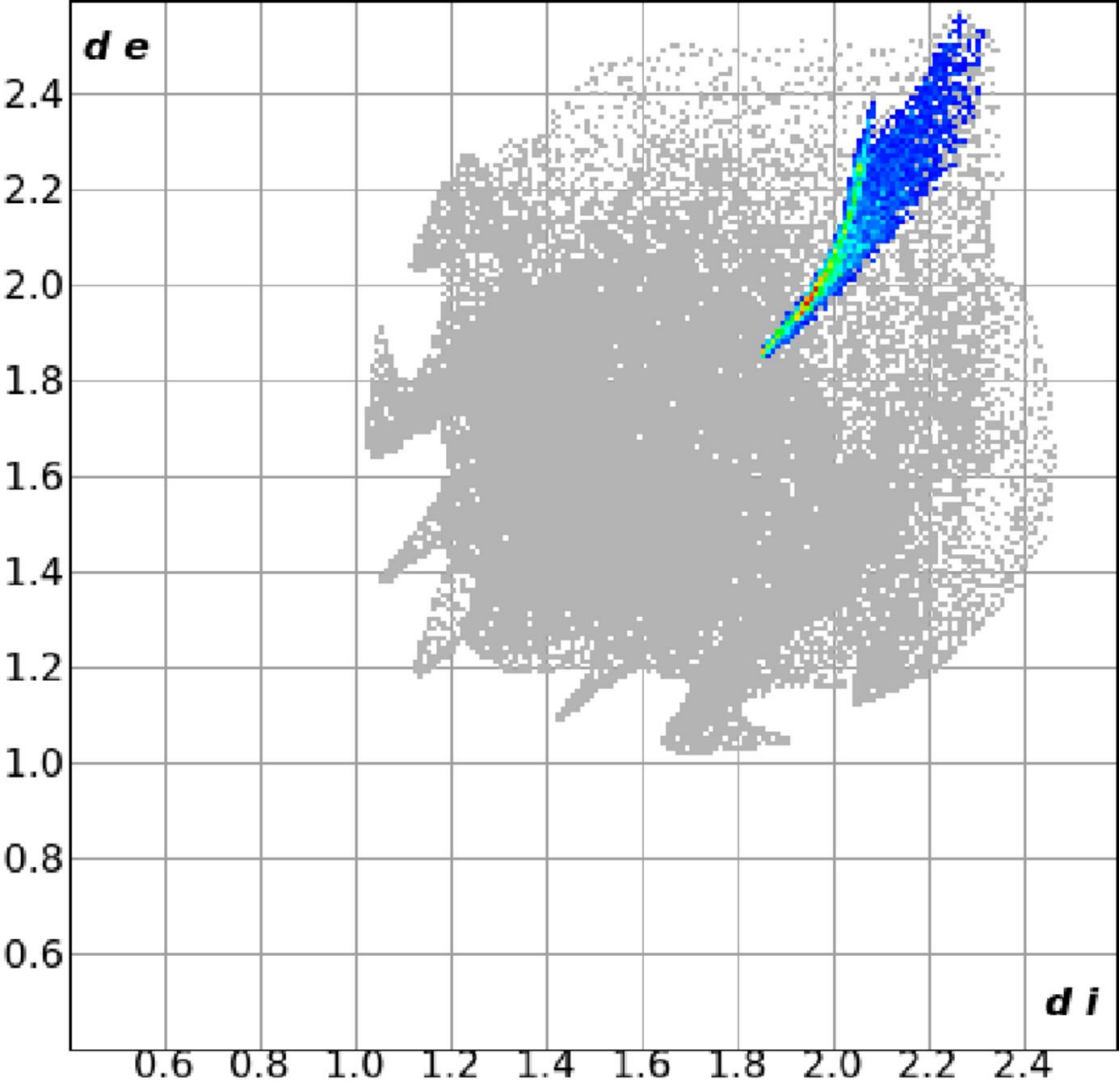
Two-dimensional fingerprint plot showing the region corresponding to inter­molecular Te⋯Te contacts in red.

The Hirshfeld surface enclosing each of the two independent mol­ecules was calculated with respect to *d*_e_, *d*_i_ and *d*_norm_ using the *Crystal Explorer* program (Spackman *et al.*, 2021 ▸), where *d*_e_ and *d*_i_ represent the nearest distance of external or inter­nal nucleus from a point of inter­est on the iso-surface. The surfaces of both independent mol­ecules are nearly identical, indicating inter­actions as bright-red areas on the Hirshfeld surface as shown in Fig. 2 ▸. The strongest of these corresponds to the Te1⋯Te2 close contact of 3.7191 (4) Å between the tellurium atoms in the two independent mol­ecules (Fig. 1 ▸). This compares to 2.7072 (9) Å for the representative covalent Te—Te bond of diphenyl ditelluride (Fuller *et al.*, 2010 ▸), indicating a relatively weak inter­action. A two-dimensional fingerprint plot highlighting the reciprocal Te⋯Te contact is shown in Fig. 3 ▸: it accounts for 8.8% of the surface area.

*checkCIF* (Spek, 2020 ▸) reports a 100% fit for an inversion center in the title structure and suggests space group *Pccn*. Indeed, the structure can be solved and refined in *Pccn* with *Z*′ = 1, but the *R*(*F*) value is 0.18, the ellipsoids are elongated, and there are numerous violations of all three glide-plane absence conditions. The *P*2_1_2_1_2 refinement yields a local center at 0.740, 0.758, 0.749, offset from the position necessary for *Pccn*, so it is clear that this is a case of the ‘inverse Marsh’ situation (Fronczek, 2018 ▸), where the structure can be approximately described in a space group of too high symmetry.

## Synthesis and crystallization

**Figure 4:**
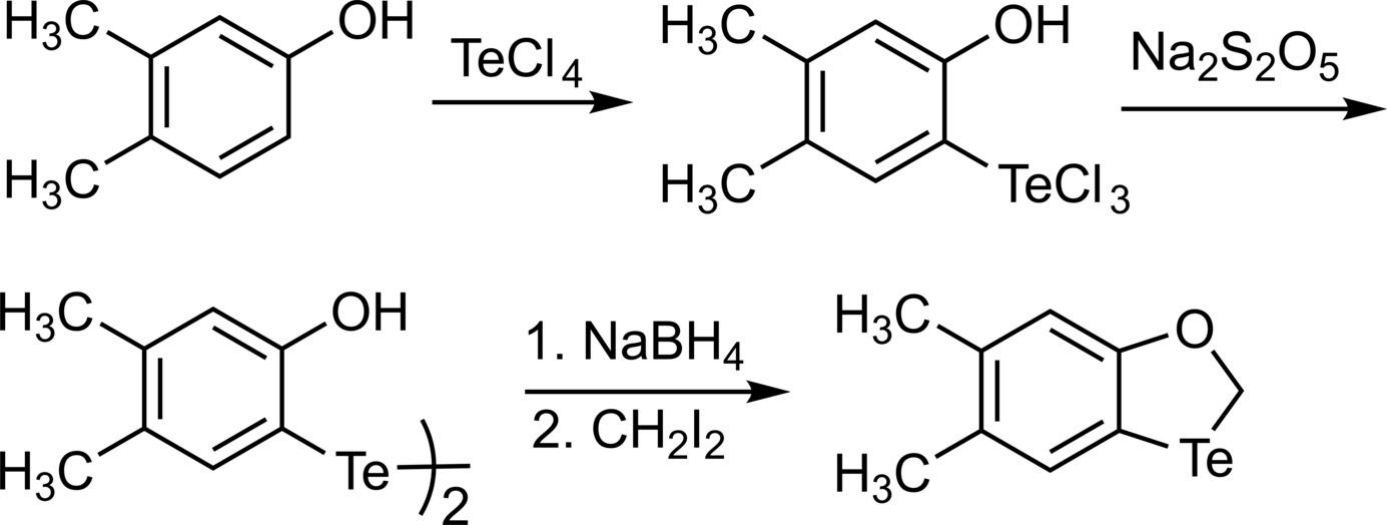
Synthesis of the title compound.

The title compound was prepared in three steps, starting with 3,4-di­methyl­phenol and tellurium tetra­chloride as outlined in Fig. 4 ▸.

**2-Hy­droxy-3,4-di­methyl­tellurium trichloride:** A 100 ml round-bottom flask with magnetic stirring, reflux condenser and drying tube was charged with tellurium tetra­chloride (4.31 g, 16 mmol), 3,4-di­methyl­phenol (1.95 g, 16 mmol) and dry toluene (8 ml). The mixture was stirred and heated to reflux for 45 min. A color change to dark yellow was observed. The clear solution was deca­nted from solids (mostly tellurium) while still hot and allowed to cool. The resulting product was collected by filtration. Yellow crystals, 2.77 g (49%). The product was pure enough for further use. An analytical sample was obtained by recrystallization from aceto­nitrile, m.p. 174–175°C, ^1^H NMR (DMSO-*d*_6_, p.p.m.): 2.18 (*s*, 3H); 2.21 (*s*, 3H) 6.78 (*s*, 1H), 7.62 (*s*, 1H). ^13^C NMR (CDCl_3_, p.p.m.): 18.68, 19.53, 117.00, 128.44, 130.70, 141.85, 154.33.

**Bis(2-hy­droxy-4,5-di­methyl­phen­yl) ditelluride:** A 100 ml round-bottom flask with magnetic stirring was charged with 2-hy­droxy-3,4-di­methyl­tellurium trichloride (2.13 g, 6 mmol), sodium metabisulfite (3.42 g, 18 mmol), 95% ethanol (2 ml), water (10 ml) and di­chloro­methane (10 ml). The mixture was stirred for 5 min, during which time it turned dark red. It was subjected to centrifugation to achieve phase separation. The organic phase was collected with a pipette and the solvent evaporated as quickly as feasible under reduced pressure. Red solid, 0.72 g (48%), mp ∼310°C (decomposition). The product is stable in solid form but decomposes rapidly in solution with tellurium formation. Consequently, it was not characterized by NMR spectroscopy.

**5,6-Di­methyl­benzo[** ***d*** **][1,3]oxatellurole:** A 50 ml round-bottom flask with magnetic stirring, reflux condenser and nitro­gen purge line was charged with bis­(2-hy­droxy-4,5-di­methyl­phen­yl) ditelluride (0.24 g, 0.5 mmol) and 95% ethanol (5 ml). The mixture was purged with nitro­gen and excess sodium borohydride was added (80 mg, 2 mmol). The mixture was stirred for 5 min, then brought to reflux for 2 min to assure complete reduction. Di­iodo­methane was added (0.2 g, 0.75 mmol) and heating resumed for another 5 min, resulting in a color change to yellow. The product subsequently precipitated after addition of water (15 ml) and was collected by centrifugation. It was taken up in chloro­form (5 ml), the solution centrifuged to remove traces of solids and the product crystallized by concentration to approx. 1 ml volume. Yellow needles, 61 mg (23%), m.p. 159–160°C. Like other monotellurides, the product is prone to slow oxidation in solution in solution. A crystal suitable for X-ray crystallography was obtained by concentration of a solution in chloro­form. ^1^H NMR (CDCl_3_, p.p.m.): 2.16 (*s*, 3H), 2.22 (*s*, 3H), 6.35 (*s*, 2H), 6.632 (*s*, 1H), 7.009 (*s*, 1 H). ^13^C NMR (CDCl_3_, p.p.m.): 19.04, 19.64, 51.11, 101.94, 112.81, 131.32, 132.93, 136.67, 159.58.

## Refinement

Crystal data, data collection and structure refinement details are summarized in Table 1 ▸. The Flack parameter (Flack & Bernardinelli, 2000 ▸) refined to 0.49 (4), indicative of an inversion twin.

## Supplementary Material

Crystal structure: contains datablock(s) I. DOI: 10.1107/S2414314623010763/hb4459sup1.cif

Structure factors: contains datablock(s) I. DOI: 10.1107/S2414314623010763/hb4459Isup2.hkl

Supporting information file. DOI: 10.1107/S2414314623010763/hb4459Isup3.mol

Supporting information file. DOI: 10.1107/S2414314623010763/hb4459Isup4.cml

CCDC reference: 2314478

Additional supporting information:  crystallographic information; 3D view; checkCIF report

## Figures and Tables

**Table 1:** Experimental details

Crystal data	
Chemical formula	C_9_H_10_OTe
*M*_r_	261.77
Crystal system, space group	Orthorhombic, *P*2_1_2_1_2
Temperature (K)	100
*a*, *b*, *c* (Å)	13.6947 (12), 23.467 (2), 5.2287 (6)
*V* (Å^3^)	1680.3 (3)
*Z*	8
Radiation type	Ag *K*α, λ = 0.56086 Å
μ (mm^−1^)	1.84
Crystal size (mm)	0.23 × 0.10 × 0.09

Data collection	
Diffractometer	Bruker D8 Venture DUO with Photon III C14
Absorption correction	Multi-scan (*SADABS*; Krause *et al.*, 2015 ▸)
*T*_min_, *T*_max_	0.701, 0.852
No. of measured, independent and observed [*I* > 2σ(*I*)] reflections	83209, 11161, 9763
*R*_int_	0.080
(sin θ/λ)_max_ (Å^−1^)	0.926

Refinement	
*R*[*F* ^2^ > 2σ(*F* ^2^)], *wR*(*F* ^2^), *S*	0.033, 0.061, 1.05
No. of reflections	11161
No. of parameters	204
H-atom treatment	H-atom parameters constrained
Δρ_max_, Δρ_min_ (e Å^−3^)	1.74, −1.28
Absolute structure	Refined as an inversion twin.
Absolute structure parameter	0.49 (4)

Computer programs: *APEX4* and *SAINT* (Bruker, 2016 ▸), *SHELXT2018/2* (Sheldrick, 2015 ▸), *SHELXL2018/1* (Sheldrick, 2015 ▸), *Mercury* (Macrae *et al.*, 2020 ▸), and *publCIF* (Westrip, 2010 ▸).

## full crystallographic data

### Crystal data

**Table:**

C_9_H_10_OTe	*D*_x_ = 2.069 Mg m^−^^3^
*M**_r_* = 261.77	Ag *K*α radiation, λ = 0.56086 Å
Orthorhombic, *P*2_1_2_1_2	Cell parameters from 9865 reflections
*a* = 13.6947 (12) Å	θ = 2.4–30.8°
*b* = 23.467 (2) Å	µ = 1.84 mm^−^^1^
*c* = 5.2287 (6) Å	*T* = 100 K
*V* = 1680.3 (3) Å^3^	Needle, yellow
*Z* = 8	0.23 × 0.10 × 0.09 mm
*F*(000) = 992	

### Data collection

**Table:**

Bruker D8 Venture DUO with Photon III C14 diffractometer	9763 reflections with *I* > 2σ(*I*)
Radiation source: IµS 3.0 microfocus	*R*_int_ = 0.080
φ and ω scans	θ_max_ = 31.3°, θ_min_ = 2.4°
Absorption correction: multi-scan (SADABS; Krause *et al.*, 2015)	*h* = −23→25
*T*_min_ = 0.701, *T*_max_ = 0.852	*k* = −43→43
83209 measured reflections	*l* = −9→9
11161 independent reflections	

### Refinement

**Table:**

Refinement on *F*^2^	Hydrogen site location: inferred from neighbouring sites
Least-squares matrix: full	H-atom parameters constrained
*R*[*F*^2^ > 2σ(*F*^2^)] = 0.033	*w* = 1/[σ^2^(*F*_o_^2^) + (0.0081*P*)^2^ + 1.4627*P*] where *P* = (*F*_o_^2^ + 2*F*_c_^2^)/3
*wR*(*F*^2^) = 0.061	(Δ/σ)_max_ = 0.001
*S* = 1.05	Δρ_max_ = 1.74 e Å^−^^3^
11161 reflections	Δρ_min_ = −1.28 e Å^−^^3^
204 parameters	Absolute structure: Refined as an inversion twin.
0 restraints	Absolute structure parameter: 0.49 (4)

### Special details

**Table:**

Geometry. All esds (except the esd in the dihedral angle between two l.s. planes) are estimated using the full covariance matrix. The cell esds are taken into account individually in the estimation of esds in distances, angles and torsion angles; correlations between esds in cell parameters are only used when they are defined by crystal symmetry. An approximate (isotropic) treatment of cell esds is used for estimating esds involving l.s. planes.
Refinement. Refined as a 2-component inversion twin.All H atoms were located in difference maps and then treated as riding in geometrically idealized positions with C—H distances 0.95 Å and with *U*_iso_(H) = 1.2*U*_eq_ for the attached C atom (0.98 Å and 1.5*U*_eq_ for methyl groups).

### Fractional atomic coordinates and isotropic or equivalent isotropic displacement parameters (Å^2^)

**Table:**

	*x*	*y*	*z*	*U*_iso_*/*U*_eq_	
Te1	0.60109 (2)	0.44282 (2)	0.70610 (4)	0.01156 (3)	
O1	0.66046 (16)	0.32225 (8)	0.7300 (5)	0.0171 (4)	
C1	0.7160 (2)	0.40889 (11)	0.9272 (6)	0.0125 (5)	
C2	0.7249 (2)	0.35032 (12)	0.8897 (6)	0.0139 (5)	
C3	0.7937 (2)	0.31871 (13)	1.0236 (6)	0.0157 (5)	
H3	0.799879	0.278965	0.993125	0.019*	
C4	0.8534 (2)	0.34532 (12)	1.2023 (8)	0.0165 (5)	
C5	0.8459 (2)	0.40437 (13)	1.2423 (6)	0.0156 (6)	
C6	0.7776 (2)	0.43536 (13)	1.1010 (6)	0.0146 (5)	
H6	0.773282	0.475424	1.124329	0.018*	
C7	0.6149 (2)	0.35744 (11)	0.5459 (6)	0.0139 (5)	
H7A	0.549569	0.342150	0.502599	0.017*	
H7B	0.654591	0.358629	0.387739	0.017*	
C8	0.9241 (2)	0.31015 (15)	1.3595 (7)	0.0229 (7)	
H8A	0.926472	0.271228	1.291820	0.034*	
H8B	0.902188	0.309218	1.537911	0.034*	
H8C	0.989287	0.327225	1.350549	0.034*	
C9	0.9089 (2)	0.43445 (15)	1.4362 (7)	0.0227 (6)	
H9A	0.901315	0.475753	1.416802	0.034*	
H9B	0.977412	0.424028	1.408941	0.034*	
H9C	0.888931	0.423143	1.608829	0.034*	
Te2	0.40868 (2)	0.43608 (2)	0.20506 (4)	0.01157 (3)	
O2	0.36896 (16)	0.31277 (8)	0.2343 (5)	0.0164 (4)	
C10	0.2997 (2)	0.39623 (11)	0.4274 (6)	0.0123 (5)	
C11	0.3004 (2)	0.33735 (11)	0.3916 (6)	0.0134 (5)	
C12	0.2362 (2)	0.30266 (12)	0.5262 (6)	0.0144 (5)	
H12	0.236775	0.262634	0.499077	0.017*	
C13	0.1713 (2)	0.32627 (12)	0.6998 (7)	0.0151 (5)	
C14	0.1696 (2)	0.38537 (12)	0.7403 (6)	0.0143 (5)	
C15	0.2342 (2)	0.41978 (12)	0.5996 (7)	0.0150 (5)	
H15	0.232865	0.459917	0.622981	0.018*	
C16	0.4082 (2)	0.35010 (11)	0.0473 (6)	0.0146 (5)	
H16A	0.367958	0.348854	−0.109910	0.018*	
H16B	0.475554	0.338379	0.002856	0.018*	
C17	0.1054 (3)	0.28707 (14)	0.8522 (7)	0.0213 (6)	
H17A	0.110824	0.248173	0.785524	0.032*	
H17B	0.037659	0.300080	0.837329	0.032*	
H17C	0.125151	0.287618	1.032360	0.032*	
C18	0.1027 (2)	0.41186 (15)	0.9348 (7)	0.0202 (6)	
H18A	0.126438	0.403030	1.107197	0.030*	
H18B	0.036663	0.396560	0.913427	0.030*	
H18C	0.101551	0.453273	0.910780	0.030*	

### Atomic displacement parameters (Å^2^)

**Table:**

	*U*^11^	*U*^22^	*U*^33^	*U*^12^	*U*^13^	*U*^23^
Te1	0.01223 (6)	0.00991 (5)	0.01252 (8)	0.00161 (5)	0.00064 (7)	0.00114 (6)
O1	0.0230 (10)	0.0110 (7)	0.0173 (12)	0.0028 (7)	−0.0044 (9)	−0.0001 (8)
C1	0.0140 (12)	0.0119 (10)	0.0115 (13)	0.0022 (9)	0.0008 (10)	0.0007 (9)
C2	0.0156 (12)	0.0142 (10)	0.0118 (12)	0.0023 (9)	0.0008 (10)	−0.0006 (9)
C3	0.0158 (12)	0.0157 (11)	0.0155 (14)	0.0036 (10)	0.0017 (11)	0.0022 (10)
C4	0.0137 (11)	0.0210 (11)	0.0147 (12)	0.0054 (9)	0.0022 (13)	0.0019 (13)
C5	0.0114 (10)	0.0239 (12)	0.0116 (15)	0.0003 (9)	0.0012 (9)	−0.0007 (9)
C6	0.0143 (11)	0.0168 (11)	0.0127 (12)	0.0006 (10)	−0.0004 (10)	−0.0021 (10)
C7	0.0161 (12)	0.0120 (10)	0.0136 (13)	0.0007 (9)	−0.0008 (10)	−0.0023 (9)
C8	0.0173 (14)	0.0296 (15)	0.0219 (17)	0.0074 (12)	−0.0014 (12)	0.0063 (13)
C9	0.0157 (12)	0.0352 (16)	0.0172 (15)	−0.0019 (14)	−0.0029 (11)	−0.0050 (13)
Te2	0.01233 (7)	0.01001 (6)	0.01239 (8)	−0.00127 (5)	−0.00103 (7)	0.00133 (6)
O2	0.0214 (9)	0.0116 (7)	0.0160 (12)	−0.0015 (7)	0.0048 (9)	−0.0006 (7)
C10	0.0132 (11)	0.0122 (10)	0.0114 (12)	−0.0020 (9)	−0.0005 (9)	0.0012 (9)
C11	0.0153 (12)	0.0122 (10)	0.0126 (12)	−0.0020 (9)	−0.0014 (10)	−0.0008 (9)
C12	0.0173 (13)	0.0140 (10)	0.0121 (13)	−0.0037 (9)	−0.0016 (11)	−0.0001 (9)
C13	0.0143 (10)	0.0174 (10)	0.0135 (12)	−0.0052 (8)	−0.0034 (12)	0.0010 (12)
C14	0.0121 (10)	0.0194 (11)	0.0115 (15)	0.0001 (9)	−0.0007 (9)	−0.0001 (9)
C15	0.0141 (12)	0.0151 (11)	0.0157 (14)	0.0001 (9)	−0.0022 (11)	−0.0011 (10)
C16	0.0189 (13)	0.0120 (9)	0.0130 (13)	−0.0009 (10)	−0.0008 (11)	−0.0021 (8)
C17	0.0200 (14)	0.0238 (13)	0.0201 (17)	−0.0080 (12)	0.0031 (12)	0.0034 (11)
C18	0.0164 (13)	0.0287 (14)	0.0157 (15)	0.0001 (12)	0.0003 (12)	−0.0025 (11)

### Geometric parameters (Å, º)

**Table:**

Te1—C1	2.108 (3)	Te2—C10	2.110 (3)
Te1—C7	2.180 (3)	Te2—C16	2.180 (3)
O1—C2	1.382 (4)	O2—C11	1.375 (4)
O1—C7	1.414 (4)	O2—C16	1.419 (4)
C1—C6	1.387 (4)	C10—C15	1.387 (4)
C1—C2	1.394 (4)	C10—C11	1.394 (4)
C2—C3	1.388 (4)	C11—C12	1.390 (4)
C3—C4	1.390 (5)	C12—C13	1.386 (5)
C3—H3	0.9500	C12—H12	0.9500
C4—C5	1.405 (4)	C13—C14	1.403 (4)
C4—C8	1.514 (4)	C13—C17	1.515 (4)
C5—C6	1.396 (4)	C14—C15	1.405 (4)
C5—C9	1.506 (4)	C14—C18	1.503 (4)
C6—H6	0.9500	C15—H15	0.9500
C7—H7A	0.9900	C16—H16A	0.9900
C7—H7B	0.9900	C16—H16B	0.9900
C8—H8A	0.9800	C17—H17A	0.9800
C8—H8B	0.9800	C17—H17B	0.9800
C8—H8C	0.9800	C17—H17C	0.9800
C9—H9A	0.9800	C18—H18A	0.9800
C9—H9B	0.9800	C18—H18B	0.9800
C9—H9C	0.9800	C18—H18C	0.9800
			
C1—Te1—C7	78.40 (11)	C10—Te2—C16	78.24 (11)
C2—O1—C7	114.5 (2)	C11—O2—C16	114.3 (2)
C6—C1—C2	118.7 (3)	C15—C10—C11	119.1 (3)
C6—C1—Te1	130.1 (2)	C15—C10—Te2	129.7 (2)
C2—C1—Te1	111.2 (2)	C11—C10—Te2	111.1 (2)
O1—C2—C3	118.9 (2)	O2—C11—C12	119.3 (2)
O1—C2—C1	119.9 (3)	O2—C11—C10	120.0 (3)
C3—C2—C1	121.0 (3)	C12—C11—C10	120.6 (3)
C2—C3—C4	119.9 (3)	C13—C12—C11	120.2 (3)
C2—C3—H3	120.1	C13—C12—H12	119.9
C4—C3—H3	120.1	C11—C12—H12	119.9
C3—C4—C5	120.0 (3)	C12—C13—C14	120.3 (3)
C3—C4—C8	119.7 (3)	C12—C13—C17	118.9 (3)
C5—C4—C8	120.2 (3)	C14—C13—C17	120.7 (3)
C6—C5—C4	118.9 (3)	C13—C14—C15	118.6 (3)
C6—C5—C9	119.7 (3)	C13—C14—C18	121.4 (3)
C4—C5—C9	121.4 (3)	C15—C14—C18	120.0 (3)
C1—C6—C5	121.4 (3)	C10—C15—C14	121.2 (3)
C1—C6—H6	119.3	C10—C15—H15	119.4
C5—C6—H6	119.3	C14—C15—H15	119.4
O1—C7—Te1	108.27 (19)	O2—C16—Te2	108.16 (19)
O1—C7—H7A	110.0	O2—C16—H16A	110.1
Te1—C7—H7A	110.0	Te2—C16—H16A	110.1
O1—C7—H7B	110.0	O2—C16—H16B	110.1
Te1—C7—H7B	110.0	Te2—C16—H16B	110.1
H7A—C7—H7B	108.4	H16A—C16—H16B	108.4
C4—C8—H8A	109.5	C13—C17—H17A	109.5
C4—C8—H8B	109.5	C13—C17—H17B	109.5
H8A—C8—H8B	109.5	H17A—C17—H17B	109.5
C4—C8—H8C	109.5	C13—C17—H17C	109.5
H8A—C8—H8C	109.5	H17A—C17—H17C	109.5
H8B—C8—H8C	109.5	H17B—C17—H17C	109.5
C5—C9—H9A	109.5	C14—C18—H18A	109.5
C5—C9—H9B	109.5	C14—C18—H18B	109.5
H9A—C9—H9B	109.5	H18A—C18—H18B	109.5
C5—C9—H9C	109.5	C14—C18—H18C	109.5
H9A—C9—H9C	109.5	H18A—C18—H18C	109.5
H9B—C9—H9C	109.5	H18B—C18—H18C	109.5

## References

[bb1] Albeck, A., Weitman, H., Sredni, B. & Albeck, M. (1998). *Inorg. Chem.* **37**, 1704–1712.

[bb2] Ba, L. A., Döring, M., Jamier, V. & Jacob, C. (2010). *Org. Biomol. Chem.* **8**, 4203–4216. 10.1039/c0Ob00086h 20714663

[bb3] Back, T. G., Kuzma, D. & Parvez, M. (2005). *J. Org. Chem.* **70**, 9230–9236. 10.1021/jo0512711 16268595

[bb4] Brodsky, M., Yosef, S., Galit, R., Albeck, M., Longo, D. L., Albeck, A. & Sredni, B. (2007). *J. Interferon Cytokine Res.* **27**, 453–462. 10.1089/jir.2007.0168 17572009

[bb5] Bruker (2016). *APEX2* and *SAINT*. Bruker AXS Inc., Madison, Wisconsin, USA.

[bb6] Carroll, P. J., Lakshmikantham, M. V., Cave, M. P., Wudl, F., Aharon-Shalom, E. & Cox, S. D. (1982). *J. Chem. Soc. Chem. Commun.* pp. 1316–1318.

[bb7] Farrar, W. V. & Gulland, J. M. (1945). *J. Chem. Soc.* pp. 11–14.

[bb8] Flack, H. D. & Bernardinelli, G. (2000). *J. Appl. Cryst.* **33**, 1143–1148.

[bb9] Fronczek, F. R. (2018). *Acta Cryst.* A**74**, a60.

[bb10] Fuller, A. L., Scott-Hayward, L. A. S., Li, Y., Bühl, M., Slawin, A. M. Z. & Woollins, J. D. (2010). *J. Am. Chem. Soc.* **132**, 5799–5802. 10.1021/ja100247y 20361797

[bb11] Groom, C. R., Bruno, I. J., Lightfoot, M. P. & Ward, S. C. (2016). *Acta Cryst.* B**72**, 171–179. 10.1107/S2052520616003954 PMC4822653 27048719

[bb12] Kojima, T., Tanaka, K., Ishida, T. & Nogami, T. (2004). *J. Org. Chem.* **69**, 9319–9322. 10.1021/jo048501g 15609977

[bb13] Krause, L., Herbst-Irmer, R., Sheldrick, G. M. & Stalke, D. (2015). *J. Appl. Cryst.* **48**, 3–10. 10.1107/S1600576714022985 PMC4453166 26089746

[bb14] Laitalainen, T., Simonen, T., Kivekäs, R. & Klinga, M. (1983). *J. Chem. Soc. Perkin Trans. 1*, pp. 333–340.

[bb15] Macrae, C. F., Sovago, I., Cottrell, S. J., Galek, P. T. A., McCabe, P., Pidcock, E., Platings, M., Shields, G. P., Stevens, J. S., Towler, M. & Wood, P. A. (2020). *J. Appl. Cryst.* **53**, 226–235. 10.1107/S1600576719014092 PMC6998782 32047413

[bb16] Persike, D. S., Cunha, R. L. O. R., Juliano, L., Silva, I. R., Rosim, F. E., Vignoli, T., Dona, F., Cavalheiro, E. A. & Fernandes, M. J. da S. (2008). *Neurobiol. Dis.* **31**, 120–126. 10.1016/j.nbd.2008.04.001 18571097

[bb17] Sheldrick, G. M. (2015). *Acta Cryst.* C**71**, 3–8.

[bb18] Spackman, P. R., Turner, M. J., McKinnon, J. J., Wolff, S. K., Grimwood, D. J., Jayatilaka, D. & Spackman, M. A. (2021). *J. Appl. Cryst.* **54**, 1006–1011. 10.1107/S1600576721002910 PMC8202033 34188619

[bb19] Spek, A. L. (2020). *Acta Cryst.* E**76**, 1–11. 10.1107/S2056989019016244 PMC6944088 31921444

[bb20] Wessig, P., John, L., Sperlich, E. & Kelling, A. (2021). *Eur. J. Org. Chem.* pp. 499–511.

[bb21] Westrip, S. P. (2010). *J. Appl. Cryst.* **43**, 920–925.

[bb22] Zigman-Hoffman, E., Sredni, B., Meilik, B., Naparstek, E. & Tartakovsky, B. (2021). *Leuk. Lymphoma*, **62**, 1146–1156. 10.1080/10428194.2020.1858292 33334225

